# Identification of a novel CDK9 inhibitor targeting the intramolecular hidden cavity of CDK9 induced by Tat binding

**DOI:** 10.1371/journal.pone.0277024

**Published:** 2022-11-15

**Authors:** Kaori Asamitsu, Takatsugu Hirokawa, Takashi Okamoto

**Affiliations:** 1 Department of Neurocognitive Science, Institute of Brain Science, Graduate School of Medical Sciences, Nagoya City University, Nagoya, Aichi, Japan; 2 Division of Biomedical Science, Faculty of Medicine, University of Tsukuba, Tsukuba, Ibaragi, Japan; 3 Transborder Medical Research Center, University of Tsukuba, Tsukuba, Ibaragi, Japan; George Mason University, UNITED STATES

## Abstract

HIV-1 transcription is specifically augmented by a transcriptional activator complex composed of Tat, an HIV-1-encoded activator, and the host transcription elongation factor P-TEFb, which is composed of cyclin-dependent kinase 9 (CDK9) and cyclin T1. Several observations suggest that P-TEFb is an attractive anti-HIV-1 drug target. However, the long-term cytotoxicity of CDK9 inhibitors hinders their widespread use in HIV-1 therapy. Thus, novel and safe inhibitors are sorely needed. By performing molecular dynamics simulations of the 3D structure of Tat/P-TEFb, we previously identified a unique cavity structure of CDK9, the CDK9 hidden cavity, that is specifically induced by Tat binding. Here, we attempted to identify compounds that fit this cavity and inhibit CDK9 activity by *in silico* screening. We identified compounds that could inhibit CDK9 activity. One of such compound, **127**, showed the strongest inhibitory activity against CDK9. Interestingly, it also inhibited CDK6 to a similar extent. We inspected the amino acid sequence and structural properties of the CDK9 hidden cavity to determine whether it is conserved in other CDKs, such as CDK6. The Ile61, comprising the center of the CDK9 hidden cavity, appears to be crucial for its kinase activity, thus indicating that the identification of the CDK9 hidden cavity may provide vital information for the development of novel CDK9 inhibitors.

## Introduction

It is estimated that more than 39 million people worldwide are living with HIV-1, with approximately 1.8 million new infections and 940,000 deaths each year (https://www.who.int/hiv/data/en/). Current combination antiretroviral therapy (cART) is considered effective in the treatment of HIV-1; however, lifelong treatment is required. In addition, chronic long-term infection with HIV-1 inevitably causes the emergence of resistant viruses and serious side effects [1, 2]. Therefore, there is a pressing need for the development of new anti-HIV-1 drugs, especially inhibitors that target processes other than those targeted by the current cART, namely HIV-1 transcription.

Transcriptional activation of the HIV-1 provirus is regulated by the virus-derived transcriptional activator protein, Tat. In the absence of Tat, there is an incomplete transcription elongation complex containing short viral transcripts of nearly 60-nucleotides, including the trans-activation response element (TAR) RNA sequence [3–5]. In the presence of Tat, a transcription activation complex containing Tat and the host positive transcription elongation factor b (P-TEFb) binds to the TAR region described above. P-TEFb contains a regulatory subunit, cyclin T1 (CycT1), and a catalytic subunit, cyclin-dependent kinase 9 (CDK9) [6–8]. CycT1 serves as the major interacting protein for Tat and specifically recruits viral mRNA [9]. CDK9 then phosphorylates the C-terminal domain of RNA polymerase II, eventually leading to a significant augmentation of viral mRNA expression [9, 10].

CDK9 was originally identified as a Tat-associated kinase (TAK) or cdc-2-like cyclin-dependent kinase PITALRE based on amino acid homology to other CDKs [11–14]. The activity of CDK9 was shown to depend on the phosphorylation of the Thr186 and Ser175 located in the regulatory "T-loop" (amino acids 168–197) of CDK9. Interestingly, Mancebo et al. [15] showed that HIV-1 replication and gene expression strictly depend on CDK9 kinase activity. Reduced levels of CycT1 and the hypophosphorylated form of CDK9 on Thr186 were preferentially observed in primary CD4(+) T cells harboring latent HIV-1, suggesting that CDK9 kinase activity is required for HIV-1 replication and gene expression in latently infected cells [16]. Upon T cell activation, where HIV-1 replication is actively ongoing, both CycT1 levels and Thr186 phosphorylation of CDK9 are greatly upregulated [16]. These observations suggest that P-TEFb is an attractive anti-HIV-1 drug target. However, the long-term cytotoxicity of current CDK9 inhibitors remains a serious obstacle to their widespread use in HIV-1 therapy [17].

In addition to phosphorylation, several studies have indicated that CDK9 activity may be modulated by conformational changes. Analysis of the crystal structure of the P-TEFb/Tat complex suggests that Tat binding induces conformational changes in CDK9, leading to alterations in its substrate specificity [18]. By performing molecular dynamics (MD) simulations, we further found that Tat induces a specific local structure in CDK9 [19]. Moreover, Wang et al. demonstrated the dynamics of the CDK9-CycT1 interface and ATP pocket reorganization upon binding by different Tat mutants [20]. These observations indicate that Tat regulates CDK9 activity by inducing conformational changes in the CDK9 "catalytic cavity" and the recruitment of the Tat/P-TEFb transcriptional activation complex in the vicinity of TAR, which may initiate transcriptional elongation in latently infected cells.

Recently, MD simulations of X-ray 3D structures have shown that local conformational changes occur within molecules, which may function as drug target cavities. For example, Filomia et al. [21] decoded hidden intramolecular cavities compatible with effective inhibitor chemicals using MD simulations of MAP kinase p38. In the present study, we exploited this hidden catalytic cavity in CDK9 for *in silico* drug screening. We identified a chemical structure that is potentially useful for the development of specific inhibitors of HIV-1 replication.

## Materials and methods

### Structure-based in silico screening

**Fig 1:**
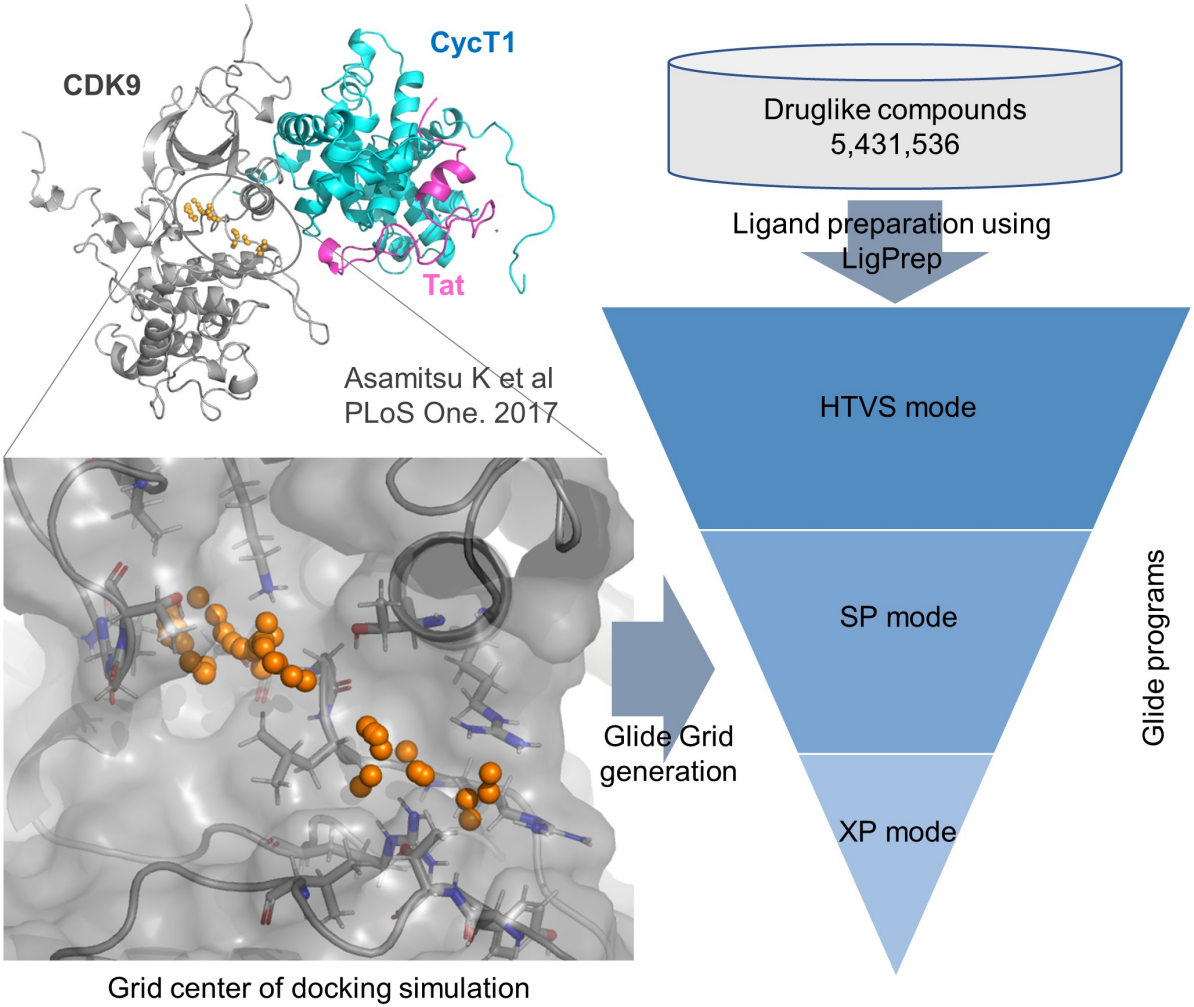
*In silico* screening work flow. Molecular docking of 5,431,536 drug-like was carried out with the Glide programs against the CDK9 hidden cavity.

We carried out *in silico* library screening based on putative druggable sites of the CDK9 hidden cavity using molecular docking against 5,431,536 drug-like [22] compounds from the Namiki HTS library set ver. 201508 (Namiki Shoji Co. Ltd.). For all the compounds, ionization and energy minimization were performed using the OPLS3 force field in the LigPrep Script of Maestro (Schrödinger, LLC). These minimized structures were used as input structures for the docking-based in silico screening. In the docking-based screening, compounds were narrowed down with progressively higher precision using three Glide [23, 24] docking programs: high-throughput virtual screening (HTVS), standard precision (SP), and extra precision (XP) (Schrödinger, LLC). A grid box was defined by small dummy atoms at the druggable site of the CDK9 hidden cavity from our previous results of the molecular dynamics simulation of the Tat/CycT1/CDK9 complex [19] (Fig 1).

### Kinase inhibition assays

#### Purification of recombinant proteins

CDK2/CycA2: Full-length human CDK2 (1–298 amino acids), accession number NP_001789.2 was co-expressed as an N-terminal GST-tagged protein with full-length GST-CyclinA2 (1–432 amino acids), accession number NP_001228.1 using the baculovirus expression system. Thereafter, GST-CDK2 was purified using glutathione sepharose chromatography.

CDK2/CycE1: Full-length human CDK2 (1–298 amino acids), accession number NP_001789.2, was co-expressed as an N-terminal GST-tagged protein with full-length CyclinE1 (1–410 amino acids), accession number NP_001229.1 using the baculovirus expression system. Thereafter, GST-CDK2 was purified using glutathione sepharose chromatography.

CDK3/CycE1: Full-length human CDK3 (1–305 amino acids), accession number NP_001249.1, was co-expressed as an N-terminal GST-fusion protein with full-length CyclinE1 (1–410 amino acids), accession number NP_001229.1 using the baculovirus expression system. Thereafter, GST-CDK3 was purified by using glutathione sepharose chromatography.

CDK4/CycD3: Full-length human CDK4 (1–303 amino acids), accession number NP_000066.1 was co-expressed as N-terminal GST-fusion protein with full-length human GST-CyclinD3 (1–292 amino acids), accession number AAA51927.1 using the baculovirus expression system. Thereafter, GST-CDK4/CycD3 was subsequently purified using glutathione sepharose chromatography.

CDK5/p25: Full-length human CDK5 (1–292 amino acids), accession number NP_004926.1, was co-expressed as an N-terminal GST-fusion protein with p25 (99–307 (end) amino acids), accession number NP_003876.1 using the baculovirus expression system. Thereafter, GST-CDK5 was purified by glutathione sepharose chromatography.

CDK6/CycD3: Full-length human CDK6 (1–326 amino acids), accession number NP_001250.1, was co-expressed as an N-terminal GST-fusion protein with full-length human GST-CyclinD3 (1–292 amino acids), accession number AAA51927.1 using the baculovirus expression system. GST-CDK6/CycD3 was purified using glutathione sepharose chromatography and activated with His-CDK7/CycH/MAT1 complex. The activated GST-CDK6/CycD3 was subsequently purified using glutathione sepharose chromatography.

CDK7/CycH/MAT1: Full-length human CDK7 (1–346 amino acids) of accession number NP_001790.1 was co-expressed as an N-terminal GST-fusion protein with full-length CyclinH (1–323 amino acids), accession number NP_001230.1, and full-length MAT1 (1–309 amino acids), accession number NP_002422.1 using the baculovirus expression system. Thereafter, GST-CDK7 was purified by using glutathione sepharose chromatography.

CDK9/CycT1: Full-length human CDK9 (1–372 amino acids), accession number NP_001252.1 was co-expressed as N-terminal GST-fusion protein with full-length His-CyclinT1 (1–726 amino acids), accession number NP_001231.2 using the baculovirus expression system. GST-CDK9 was subsequently purified using glutathione sepharose chromatography.

#### Test compounds

The test compound was dissolved in and diluted with dimethylsulfoxide (DMSO) to achieve 100-fold higher concentration. Then the solution was further 25-fold diluted with assay buffer (20 mM HEPES, 0.01% Triton X-100, 1 mM DTT, pH 7.5) to make 4x compound solution. Reference compounds for the assay control were prepared similarly.

#### Off-chip Mobility Shift Assay (MSA) and data analysis

Kinase inhibition profiles were determined using an off-chip mobility shift assay (MSA) provided by Carnabioscience (Carna Biosciences, Kobe, Japan) [25] with recombinant human CDK/cyclin complex and corresponding substrate with different ATP concentration of Km value (S1 Table). Phosphorylation of the peptide substrate in the presence and absence of the compound(s) was determined. The assay protocol was published on the Carna Biosciences website (http://www.carnabio.com/english/index.html). Briefly, 4x Substrate/ATP/Metal solution was prepared with kit buffer (20 mM HEPES, 0.01% Triton X-100, 5 mM DTT, pH7.5), and 2x kinase solution containing CDK/CycT1 complex was prepared with assay buffer. Five microliters of 4x compound solution, 5 μL of 4x Substrate/ATP/Metal solution, and 10 μL of 2x kinase solution were mixed and incubated in a well of polypropylene 384 well microplate for 1.5 or 5 hour(s) at room temperature. Reactions were stopped by the addition of 70 μL of Termination Buffer (127 mM HEPES, 0.01% Triton X-100, 26.7 mM EDTA-2Na, 1% DMSO, pH7.5). The reaction mixture was applied to the LabChip^™^ system (PerkinElmer), and the product and substrate peptide peaks were separated and quantitated. The kinase reaction was evaluated by the product ratio calculated from peak heights of the product(P) and substrate(S) peptides using the formula (P/(P+S)). The readout value of reaction control (complete reaction mixture) was set as a 0% inhibition, and the readout value of background (Enzyme(-)) was set as a 100% inhibition, then the percent inhibition of each test solution was calculated. All experiments were performed in duplicate and had a positive control for the assay verification.

### Figure preparation, similarity search, and 2D diagram analysis

Figures showing protein structures were prepared using PyMOL 2.5.2 (Schrodinger, LLC). Sequences were aligned using Clustal W. The 2D interaction of the docking pose of CDK9/**127** was analyzed by MOE ligand analysis using Molecular Operating Environment (MOE) software 2020.0901 (Chemical Computing Group).

## Results

### Detailed analysis of the CDK9 hidden cavity induced by Tat binding

**Fig 2:**
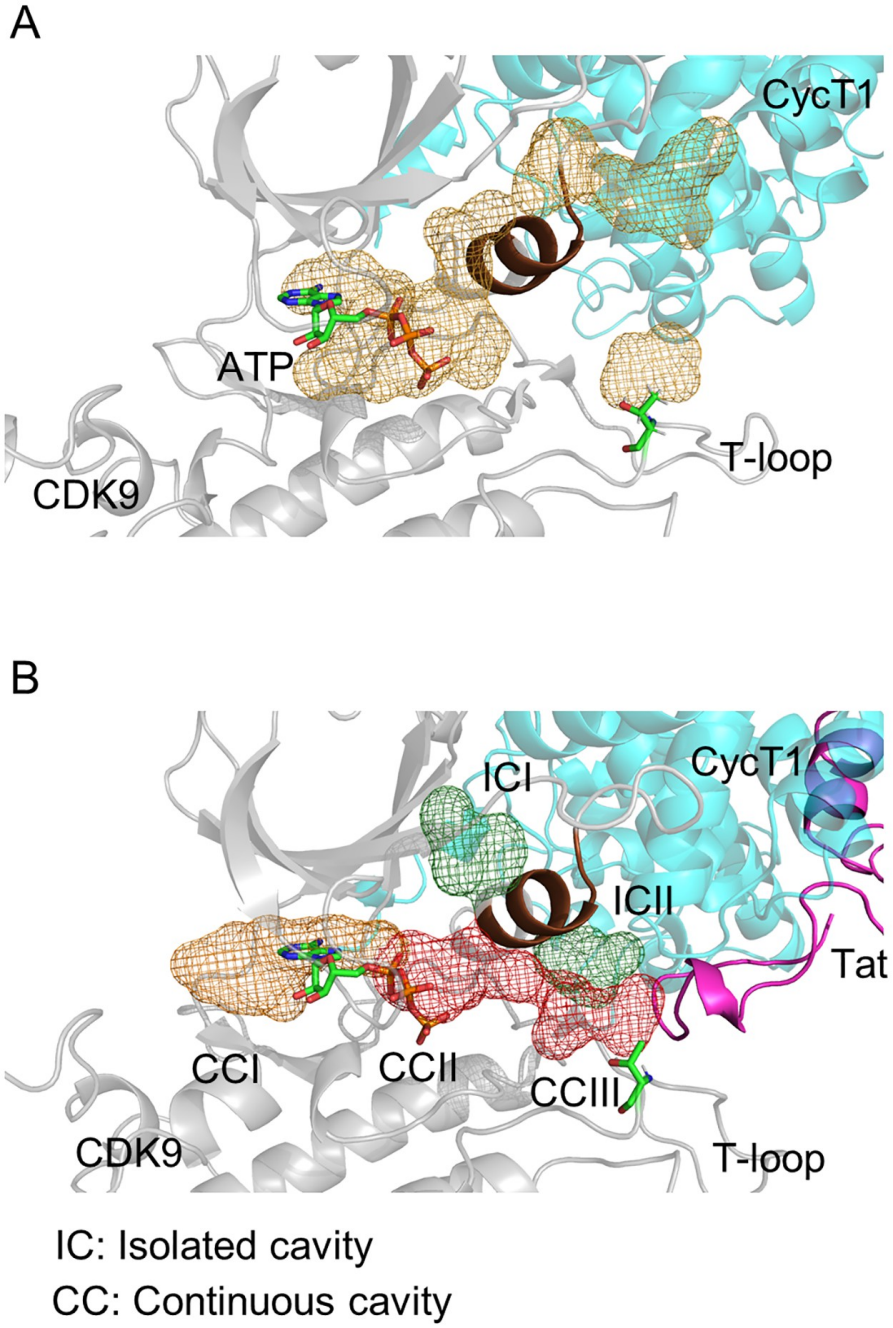
Details of the CDK9 hidden cavity induced by Tat binding. (A) Cavities on CDK9 formed with CycT1. CDK9, CycT1, and Tat are shown as gray, cyan, and pink ribbons, respectively. ATP and phosphate atoms are shown as a stick. PITALRE is shown as a brown cartoon. Cavities are shown with a mesh (orange). (B) The cavities within CDK9 induced by Tat binding, ICI and II, are shown as a green mesh. CCI–III are shown as orange and red meshes.

As previously reported [19], we performed MD simulation using the CDK9/CycT1/Tat and CDK9/CycT1 complexes, and identified a unique cavity within the CDK9 molecule. Fig 2A illustrates the CDK9 catalytic site surrounding the ATP-binding site in the CDK9/CycT1 complex in the absence of Tat. In the presence of Tat, an elongated cavity with small cavities connected to each other appeared, which we named the Tat-induced CDK9 hidden cavity (Fig 2B). We classified this cavity into three portions: continuous cavities (CCs) I, II, and III. Furthermore, we observed the dissociation of two isolated cavities, isolated cavities (ICs) I and II, apart from CCI–III. The characteristic PITALRE peptide sequence was found between ICI and II (Fig 2B). This suggests that the recruitment of Tat moves the PITALRE sequence to generate a CC between the ATP-binding site and the CDK9 catalytic center. As we previously reported [19], Tat appears to induce a structural transition of the T-loop containing Thr186 to create a substrate-binding surface in the vicinity.

### Identification of a small molecular compound, 127, that significantly inhibits CDK9 activity

Using the newly revealed cavity within CDK9, we performed *in silico* screening against the available chemical library (Namiki HTS library set ver. 201508) at a putative druggable site (hidden cavity) to identify a CDK9 inhibitor. The candidate compounds were narrowed down with progressively higher precision using three Glide docking programs: HTVS, SP, and XP (Fig 1). From a total of 5,431,536 drug-like compounds, we extracted 121,763 candidates using the HTVS mode for the next precision docking programs. These compounds were narrowed down to 121,395 by the SP mode, and 23,039 were finally selected by the XP mode.

Fifty small-molecule compounds were selected and ranked according to the docking score (S1 File). These compounds were tested for their activity against the CDK9 by an *in vitro* kinase assay using recombinant proteins of CDK9 and CycT1. We chose this assay system because Tat is a naturally denatured protein, which would make it difficult to establish a Tat-containing CDK9 *in vitro* kinase assay.

**Fig 3:**
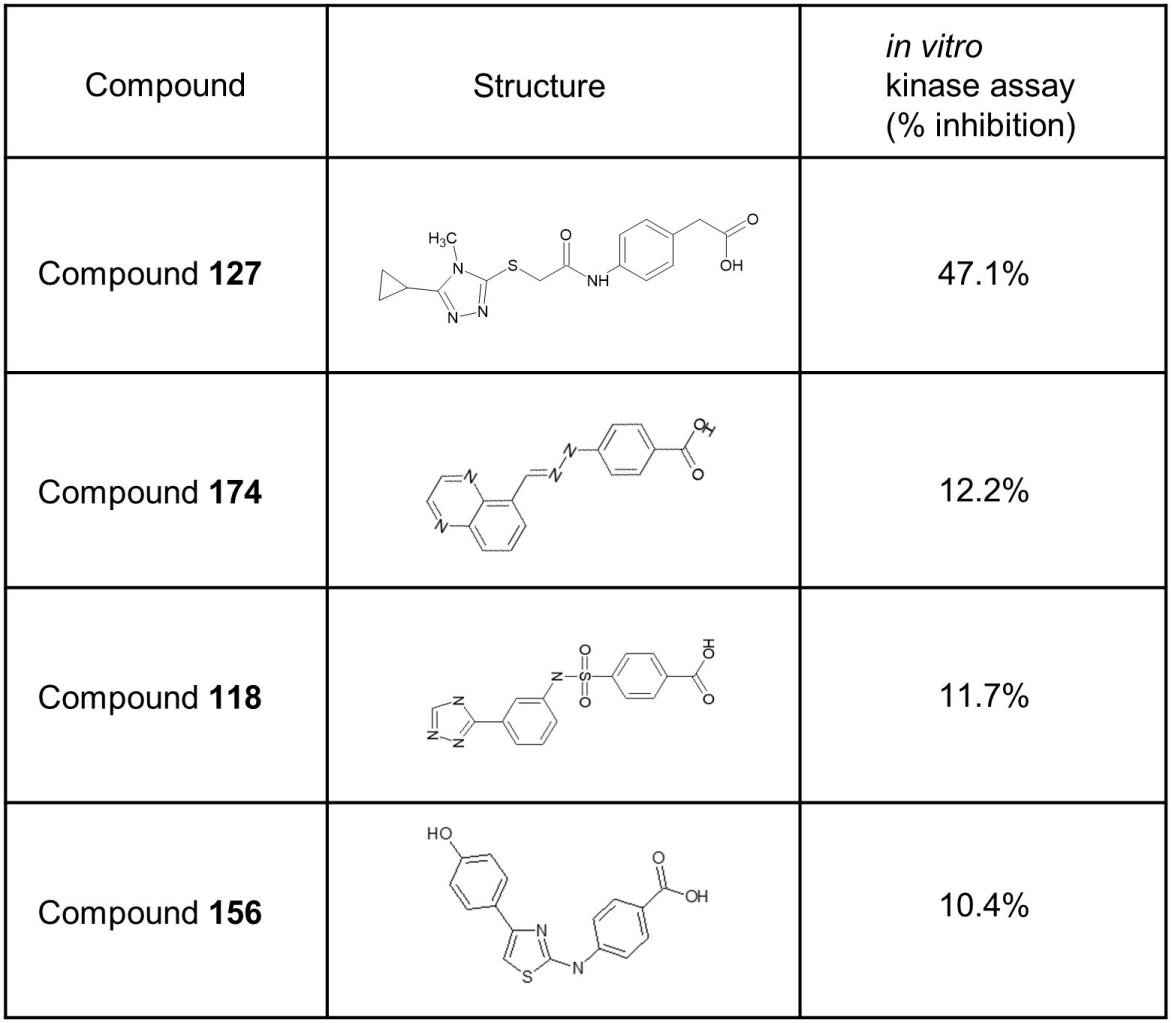
Putative CDK9 inhibitors. Four compounds showed more than 10% CDK9 inhibition in an *in vitro* kinase assay. The benzoic acid moiety is a common feature of all four compounds.

Among the 50 compounds, four showed greater than 10% inhibitory effects on CDK9 activity at 10 μM (Fig 3 and S2 File). Fig 3 depicts their chemical structures and the extent of CDK9 inhibition *in vitro*. All compounds had a carboxyl group at the *para* position of the benzene ring, which occupied the CCII-III cavity of CDK9. It was noted that only compound **127**, which exhibited significant inhibition of CDK9, had a CH2 linker between the benzene ring and the carboxyl group, suggesting that the CH2 linker enhanced the fitting between the compound and the hidden cavity. The structure on the left side of **127** is also different from that of the other compounds, which may be responsible for its higher binding.

**Fig 4:**
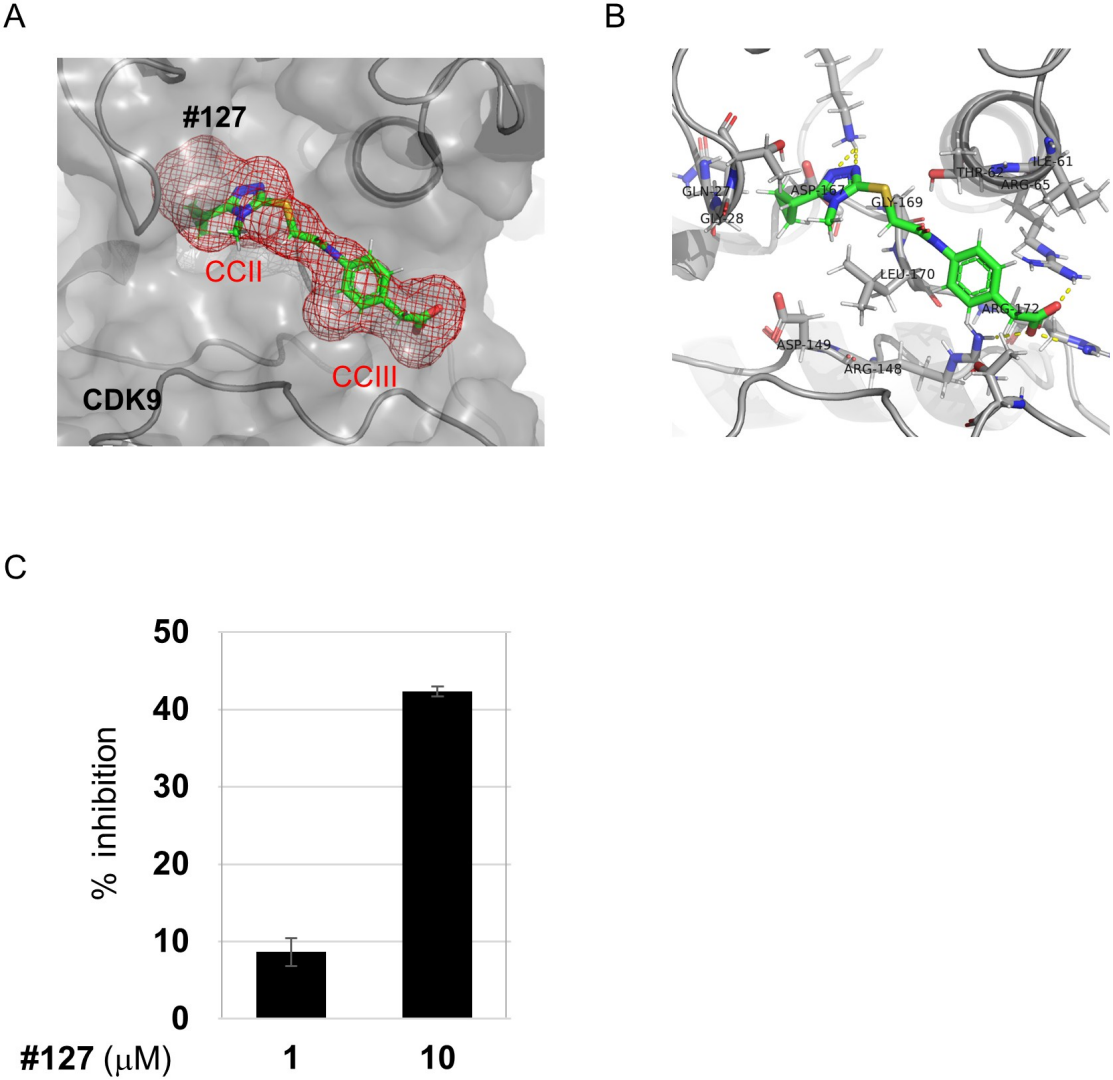
The novel CDK9 inhibitor, compound 127. (A) The predicted binding mode of **127** and CCII and III (red mesh surface) within the CDK9 hidden cavity. (B) Key residues surrounding **127** at the distance of 4.5 Å. (C) The dose-dependent inhibition of CDK9 activity by **127**. Relative kinase activity is shown as % inhibition as described in the Off-chip Mobility Shift Assay (MSA) and data analysis section. Results are shown as means ± SD from 4 independent experiments (n = 4).

The docking pose of the CDK9/**127** complex is shown in Fig 4A and 4B. Compound **127** consists of two parts, triazole and benzoic acid, which interact with CCII and III, respectively. Key residues surrounding **127** at a distance of 4.5 Å were detected (Fig 4B) and defined as the **127**-associated local structure. This structure was composed of 19 amino acids: Gln27, Gly28, Thr29, Val33, Lys48, Ile61, Thr62, Arg65, Arg148, Asp149, Lys151, Asn154, Asp167, Gly169, Leu170, Arg172, Tyr185, Thr186, and Val189. This docking model suggests that the carboxyl group of **127** forms an ionic bond with the side chains of Arg65, Arg148, and Arg172, and the triazole and benzene rings are in close contact with the side chains of Leu170 and Ile61, respectively. As shown in Fig 4C and S3 File, **127** dose-dependently inhibited CDK9.

### The effect of 127 on the activity of various CDKs

To identify the specificity of **127** for CDK9, we performed an *in vitro* kinase assay using various CDKs and their counterpart cyclins (Table 1 and S4 File). Compound **127** significantly inhibited CDK6, and its inhibitory effect was stronger than that on CDK9. Thus, we investigated the structural similarities among various CDKs in terms of full-length CDKs and the **127**-associated local structures, namely global AA similarity and local AA similarity, respectively.

**Table 1:** Inhibitory effects of compound 127 on various CDKs.

Kinase	Inhibition (%) 127 (10 μM)
CDK2/CycA2	5.4
CDK2/CycE1	6.9
CDK3/CycE1	15.6
CDK4/CycD3	15.1
CDK5/p25	−5.9
CDK6/CycD3	81.3
CDK7/CycH/MAT1	−4.3
CDK9/CycT1	40.9

Sequence aliment of full-length CDKs and **127**-associated local structures were performed using Clustal W (S1 Fig). As shown in Table 2, the local AA similarity of CDKs to CDK9 ranged from 68% to 90%, whereas the global AA similarity of CDKs to CDK9 ranged from 26% to 37%. The conservation of global AA similarity or local AA similarity between CDK6 and CDK9 did not differ from that between CDK9 and other CDKs. These observations indicated that the specificity of **127** was not associated with the primary AA sequence. Thus, we hypothesized that the nature of the amino acid residues, especially their side chains, constituting the local structure of CDK9 for interaction with **127** might determine the specificity of CDK inhibition. Therefore, protein–ligand interactions were analyzed to reveal significant amino acid residues through functional interactions with the ligand.

**Table 2:** Sequence similarity of full-length CDKs (global AA similarity) and the amino acid residues constituting the local structure on CDK9 surrounding 127 (local AA similarity).

Local AA similarity								
	CDK2	CDK3	CDK4	CDK5	CDK6	CDK7	CDK8	CDK9
CDK2		100	73.7	89.5	78.9	84.2	68.4	89.5
CDK3	74.3		73.7	89.5	78.9	84.2	68.4	89.5
CDK4	44.6	45.05		73.7	89.5	68.4	73.7	78.9
CDK5	59	60	43.4		78.9	73.7	73.7	78.9
CDK6	46.6	45.4	67.15	43.8		73.7	73.7	73.7
CDK7	40.6	40.75	34.35	42.3	31.85		57.9	78.9
CDK8	31.15	32.3	30.25	31.55	28.2	29.5		68.4
CDK9	35.95	35.5	32.65	36.7	32.25	33.75	25.95	
Global AA similarity								

**Fig 5:**
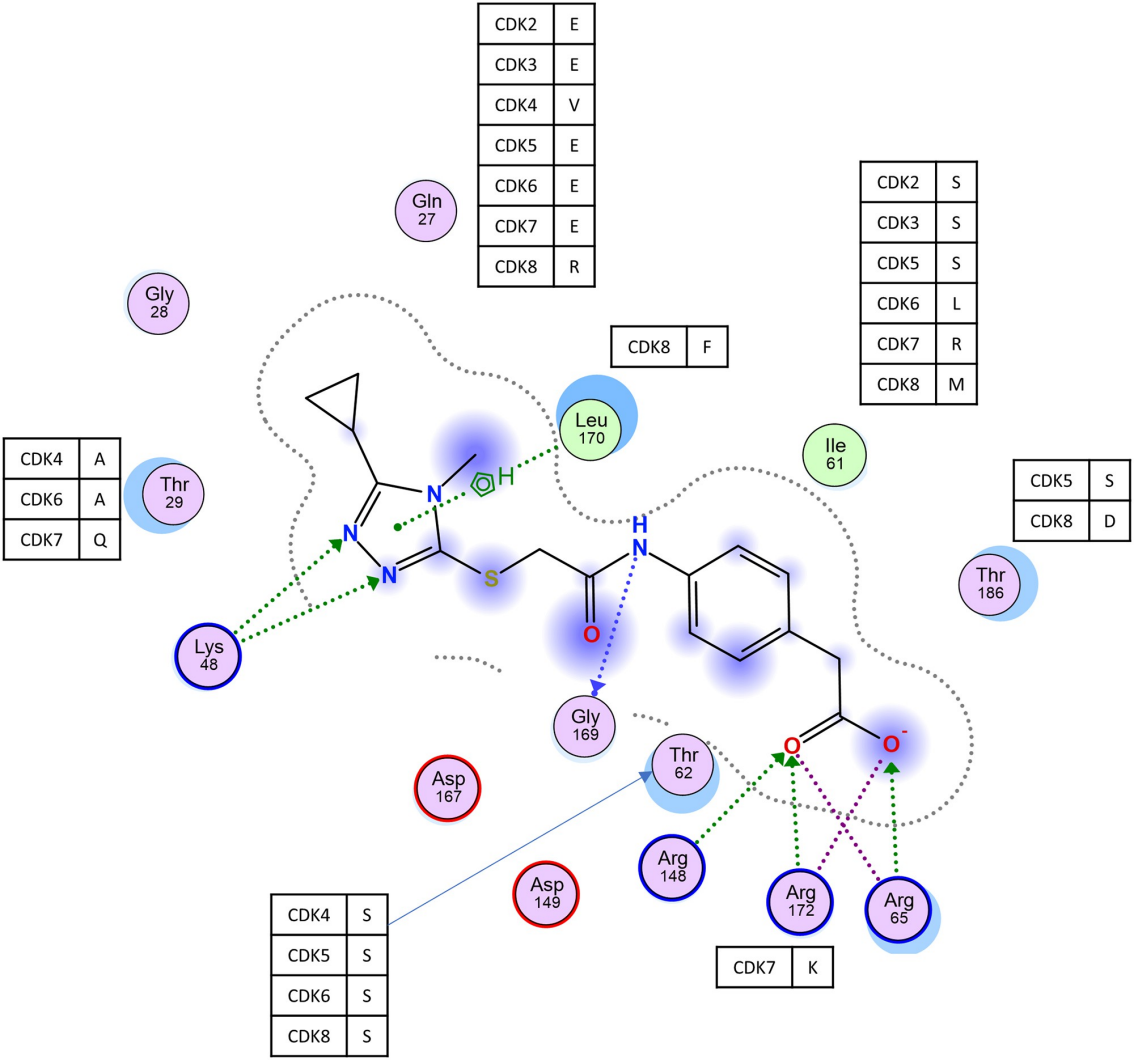
A 2D interaction diagram of the docking pose of 127 in the ligand-binding site of CDK9. Hydrophobic and polar amino acids are shown in green and purple circles, respectively. Interactions with the amino acids are depicted as green dotted arrows (side chain acceptor) or green dotted lines (aromatic). Amino acid variations among CDKs are shown in the figure.

As shown in Fig 5, 14 AA residues exhibiting contact with **127** were detected within the CDK9 protein by MOE ligand analysis. These residues were contained in the **127**-associated local structure mentioned earlier. Seven amino acid residues, Gly28, Lys48, Arg65, Arg148, Asp149, Asp167, and Gly169, were completely conserved among various CDKs (Fig 5). Among other residues, two amino acids residues, Gln27 and Ile61, were highly polymorphic (Fig 5). Gln27 is shown to form hydrogen bonds and possibly electrostatic interactions with **127**. The residue of CDK6 corresponding to this amino acid residue is Glu, which has similar properties to those of Gln, suggesting that Gln27 may be irrelevant in determining the CDK specificity of **127**. Interestingly, the CDK9 Ile61 counterpart in CDK6 is Leu; these are very similar amino acids, whereas other CDKs have charged AAs. Since this hydrophobic interaction is formed in close vicinity to the area of ionic interactions between the positively charged AAs of CDK9, Arg65, Arg148, and Arg172, and the two oxygen atoms of **127**, it may elicit a strong interaction between **127** and CDK9. However, CDK4 has the same AA at this position, but **127** shows no inhibitory activity. Thus, it is likely that other amino acid differences, such as Gln27 and Thr62 (in CDK9), might be involved in the **127**-mediated inhibition.

### The substructure of 127 is important for CDK9 inhibition

**Fig 6:**
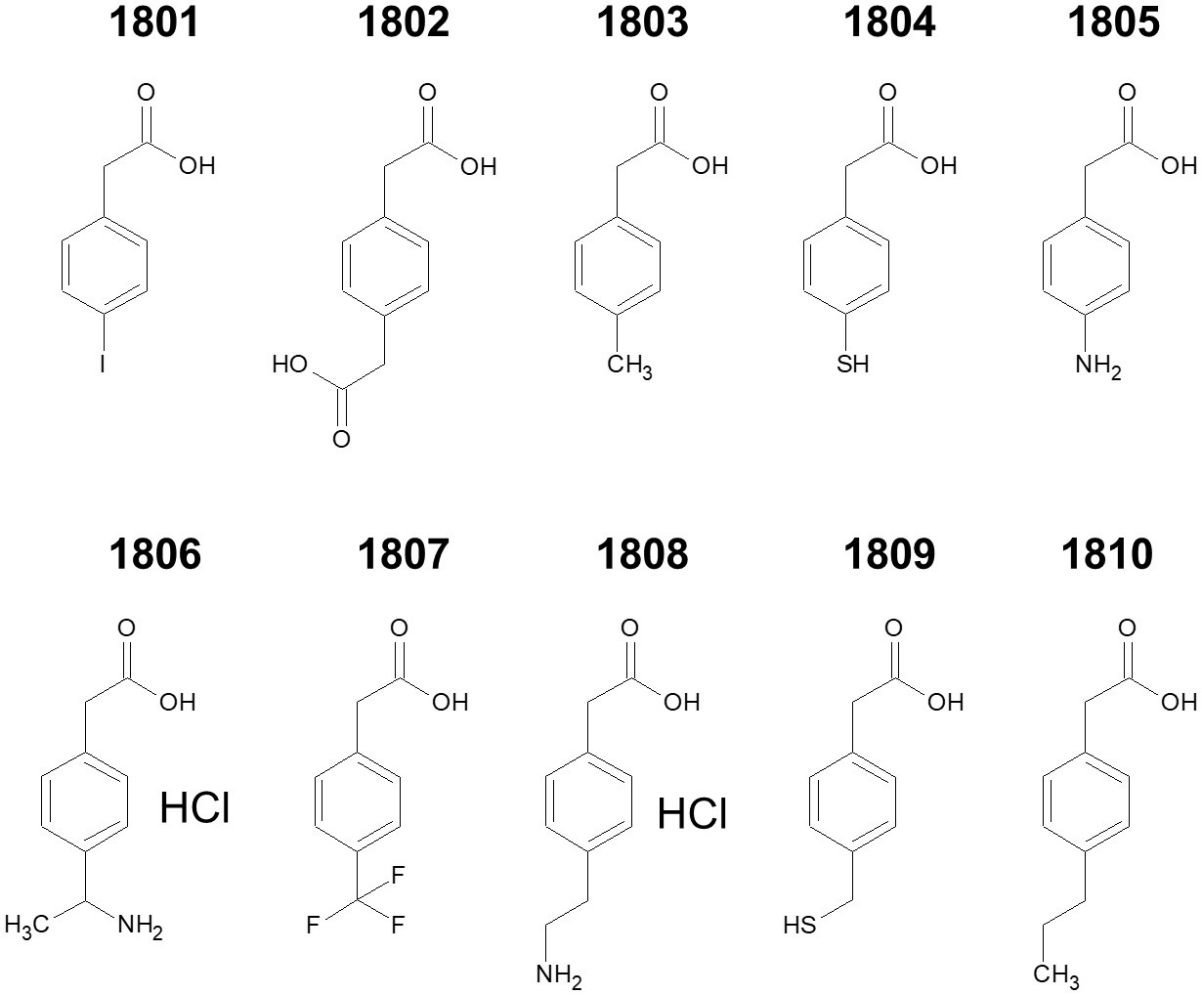
Substructure derivatives of 127. A total of 10 compounds containing the benzoic acid moiety were purchased and their CDK9 inhibitory activity was examined.

Since chemical moieties other than benzoic acid are quite heterogeneous among the candidate CDK9 inhibitors obtained in this study, we tested the efficacy of various compounds related to this structure. As depicted in Fig 6, we obtained such compounds and tested their CDK9 inhibitory effects *in vitro*. Compounds **1801**, **1804**, and **1805** significantly inhibited CDK9 (Table 3, S3 and S5 Files). Among these compounds, **1804** exhibited the strongest inhibition, although **1805** was a direct derivative of **127**. These compounds were only partial derivatives of the candidate CDK9 inhibitor; therefore, little inhibitory effect was observed at 10 μM concentration. Since **1804**, **1805**, and **1806** act as electron-withdrawing compounds from their benzene rings, it is possible that this characteristic is involved in the CDK9-inhibiting activity. However, since the inhibitory extent of compounds **1808** and **1809** was significantly lower, there might be some distance effect between the benzene ring and these positively charged residues.

**Table 3:** Inhibitory effects of partial compounds on CDK9.

	CDK9 inhibition (%)	
Compound	10 μM	500 μM
1801	4.1	43.6
1802	1.5	23.2
1803	0.8	-5.8
1804	0.6	88.3
1805	1.2	47.7
1806	1.7	28.6
1807	-1.9	6.2
1808	0.6	-5
1809	1.9	15.2
1810	2.2	3.8

Finally, we examined the inhibitory effects of **1804** on the other CDKs. In addition to CDK6 and CDK9, this compound also inhibited several other CDKs (Table 4 and S6 File). This can be explained by the similarity in the AA composition of each CDK to that of CDK9. Unexpectedly, **1804** exhibited a strong inhibitory effect on CDK7/CycH/MAT1, which is distinct from the result obtained with **127**, suggesting the possible induction of the target by the SH moiety of **1804**. This information is valuable for the development of specific and effective inhibitors of each CDK.

**Table 4:** Inhibitory effects of compound 1804 on various CDKs.

Kinase	Inhibition (%) 1804 (500 μM)
CDK2/CycA2	62.4
CDK2/CycE1	46.3
CDK3/CycE1	59.3
CDK4/CycD3	45.4
CDK5/p25	35.6
CDK6/CycD3	69.5
CDK7/CycH/MAT1	86.4
CDK9/CycT1	76.9

## Discussion

Current cART is effective at managing HIV-1 infection but does not cure the disease. Chronic long-term infection with HIV-1 causes the emergence of resistant viruses and serious side effects [1, 2]. Thus, it is important to develop the development of new HIV-1 drugs is sorely needed. P-TEFb, which is composed of CycT1 and CDK9, is an attractive anti-HIV-1 drug target. However, the long-term cytotoxicity of current CDK9 inhibitors hinders their widespread use in HIV-1 therapy [17]. Tat induces a specific local structure in CDK9, and may regulate CDK9 activity through such conformational changes [19]. Here, we sought to determine whether this local structure could function as a drug target cavity.

We performed *in silico* screening for novel CDK9 inhibitors using the hidden cavity [19] that emerged upon MD simulation of the Tat/CycT1/CDK9 trimolecular complex. This cavity is formed upon the binding of Tat to the P-TEFb complex, thus serving as a good pharmacophore for effective inhibitors. As demonstrated above, we identified several interesting compounds that exhibited significant inhibitory effects against CDK9. We used an *in vitro* kinase system composed of purified recombinant CDK9 and CycT1 proteins. The catalytic activity was monitored by the extent of the activity on synthetic peptide substrates optimized for CDK9 activity, as measured by the MSA method [25]. We admit that this simple assay system may have overlooked specific CDK9 inhibitor compounds because we did not incorporate Tat or the authentic substrate peptide derived from RNA pol II. Nevertheless, analyzing the compounds obtained in this assay allowed us to demonstrate that the hidden CDK9 cavity is real. Because this site is different from the authentic site, there is the potential to obtain CDK inhibitor compounds that are quite different from compounds with known side effects and low specificity.

In recent years, "block and lock" therapy, which reinforces the latent state of HIV-1, has been proposed as a new therapeutic approach for its HIV-1 eradication [26]. The agents used in this therapy are particularly important to achieve sustained cART-free HIV-1 remission. Such compounds target the transcriptional activation of HIV-1 by Tat, and several compounds are currently under development. For example, didehydro-cortistatin A is thought to enhance latency by suppressing the epigenetic environment of the HIV-1 LTR, resulting in stable and prolonged mobilization of histone deacetylation and transcriptional repression complexes [27]. Additional compounds such as sudemycin D6 and ZL-0580 also target host factors upstream of the Tat/P-TEFb interaction and continue to extend the latency after withdrawal [28, 29]. CDK9 inhibitors may also affect the maintenance of HIV-1 latency, and the compounds found in this study may function in this manner.

CDK9 and P-TEFb have also emerged as candidate targets in cancer and leukemia because of their critical role in regulating gene expression [30], and it has been suggested that CDK9 also reactivates tumor suppressor genes and is involved in promoting and maintaining cancer cell growth [30]. Several clinical trials testing CDK9 inhibitors are currently underway in advanced solid and hematologic malignancies [31]. If more specific and potent CDK9 inhibitors are developed as an extension of our current study, they will lead to the development of novel anticancer therapies with better pharmaceutical profiles.

CDK9 inhibitors that are effective in the treatment of AIDS and cancer must be taken long-term and must have high specificity. However, to date, no compound satisfying these requirements has been developed. This is thought to be because most CDK9 inhibitors target the ATP-binding domain [32]. In the present study, we targeted the CDK9 hidden cavity, which consists of a region distinct from the ATP-binding pocket of CDK9. However, the obtained compounds showed inhibitory effects on other CDKs, suggesting that the CDK9 hidden cavity is conserved and universally functional in other CDKs. The modification and application of the CDK9-like hidden cavity to other CDKs may lead to the development of novel inhibitors for the respective CDKs. Thus, similar approaches are warranted for each CDK.

P-TEFb is also used by other viruses, including human T-lymphotropic virus (HTLV-1), herpes simplex virus (HSV-1 and HSV-2), human cytomegalovirus (CMV), Epstein-Barr virus (EBV), human adenovirus, influenza A virus, dengue virus, and Kaposi sarcoma-associated virus (KSHV) [33]. Most viruses hijack P-TEFb through physical interactions with virus-encoded proteins to facilitate efficient transcription of the viral genome. Our CDK9 development strategy for targeting the specific local structure of P-TEFb and viral protein complexes may be useful for the development of novel therapeutics against other viral diseases involving P-TEFb.

## Supporting information

S1 Fig Sequence comparison of CDK9 and other CDKs. Amino acid alignments of CDKs were performed using Clustal W. The key amino acid residues constituting the local structure surrounding **127** are shown in boxes.
(TIF)

S1 Table Reaction conditions of *in vitro* kinase assay. (DOCX)

S1 File Structures and docking scores of 50 compounds identified using in silico screening chosen for *in vitro* CDK9 kinase assay. The docking score was calculated using extra precision (XP) of Glide docking programs and shown in kcal/mol.
(PDF)

S2 File The raw data of Fig 3. (XLSX)

S3 File The raw data of Fig 4 and Table 3. (XLSX)

S4 File The raw data of Table 1. (XLSX)

S5 File The raw data of Table 3. (XLSX)

S6 File The raw data of Table 4. (XLSX)
