# Supplementary figures and images for: Identification of a novel CDK9 inhibitor targeting the intramolecular hidden cavity of CDK9 induced by Tat binding
Source: PLoS One. 2022 Nov 15;17(11):e0277024. doi: 10.1371/journal.pone.0277024 (PMC9665388; doi:10.1371/journal.pone.0277024)

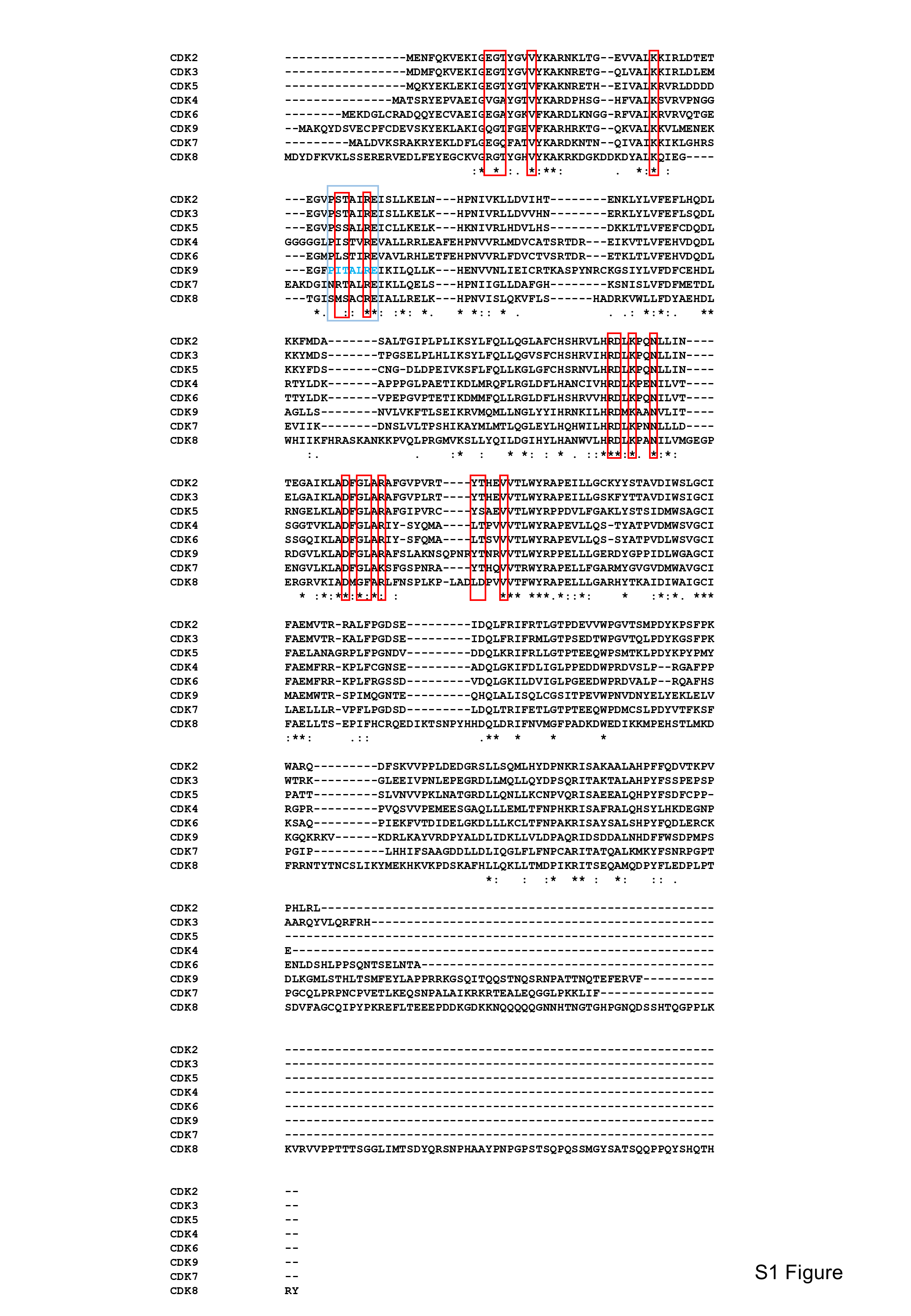

Supplement: S1 Fig — Amino acid alignments of CDKs were performed using Clustal W. The key amino acid residues constituting the local structure surrounding 127 are shown in boxes. (TIF) [file pone.0277024.s001.tif]
