# Supplementary material for: Identification of a novel CDK9 inhibitor targeting the intramolecular hidden cavity of CDK9 induced by Tat binding
Source: PLoS One. 2022 Nov 15;17(11):e0277024. doi: 10.1371/journal.pone.0277024 (PMC9665388; doi:10.1371/journal.pone.0277024)
Supplement: S1 Table — (DOCX) [file pone.0277024.s002.docx]

**S1 Table. Reaction conditions of in vitro kinase assay.**

| Kinase | | Substrate | | ATP | Metal | | Incubation time (hrs) | Positive control |
| --- | --- | --- | --- | --- | --- | --- | --- | --- |
| Name | Conc.(ng/ml) | Name | Conc. (nM) | Conc. (μM) | Name | Conc. (mM) |  |  |
| CDK2/CycA2 | 40 | Modified Histone H1 | 1000 | 25 | Mg | 5 | 1.5 | Staurosporine |
| CDK2/CycE1 | 20 | Modified Histone H1 | 1000 | 150 | Mg | 5 | 1.5 | Staurosporine |
| CDK3/CycE1 | 50 | Modified Histone H1 | 1000 | 1000 | Mg | 5 | 1.5 | Staurosporine |
| CDK4/CycD3 | 120 | DYRKtide-F | 1000 | 200 | Mg | 5 | 5 | Staurosporine |
| CDK5/p25 | 10 | Modified Histone H1 | 1000 | 10 | Mg | 5 | 1.5 | Staurosporine |
| CDK6/CycD3 | 250 | DYRKtide-F | 1000 | 30 | Mg | 5 | 5 | Staurosporine |
| CDK7/CycH/MAT1 | 800 | CTD3 peptide | 1000 | 50 | Mg | 5 | 5 | Staurosporine |
| CDK9/CycT1 | 1000 | CDK9 substrate | 1000 | 10 | Mg | 5 | 5 | Staurosporine |
