# Supplementary material for: Identification of a novel CDK9 inhibitor targeting the intramolecular hidden cavity of CDK9 induced by Tat binding
Source: PLoS One. 2022 Nov 15;17(11):e0277024. doi: 10.1371/journal.pone.0277024 (PMC9665388; doi:10.1371/journal.pone.0277024)
Supplement: S1 File — The docking score was calculated using extra precision (XP) of Glide docking programs and shown in kcal/mol. (PDF) [file pone.0277024.s003.pdf]

ID: 10

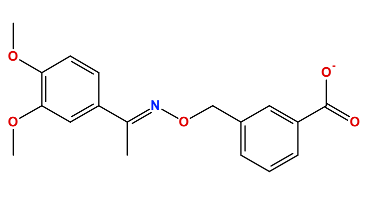

Docking Score: -5.9300

ID: 16

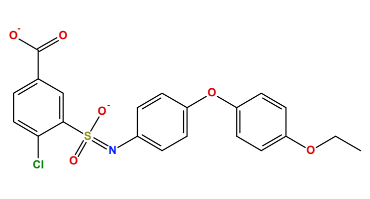

Docking Score: -5.8237

ID: 29

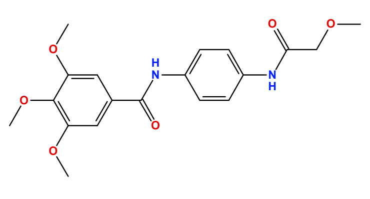

Docking Score: -5.1698

ID: 30

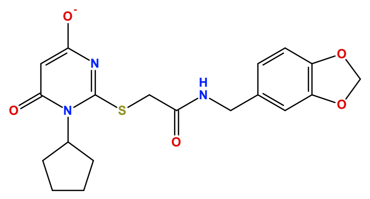

Docking Score: -4.3903

ID: 31

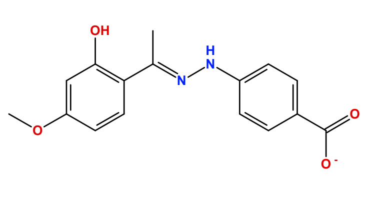

Docking Score: -6.2186

ID: 34

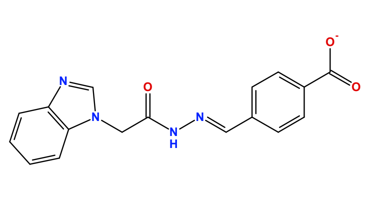

Docking Score: -5.2326

ID: 35

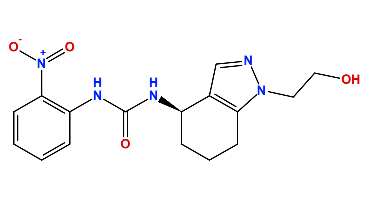

Docking Score: -4.6572

ID: 39

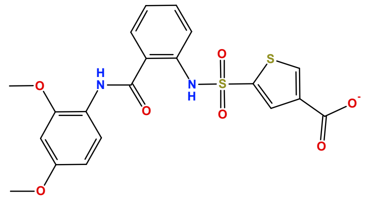

Docking Score: -5.6467

ID: 40

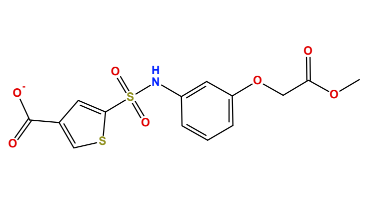

Docking Score: -7.7741

ID: 42

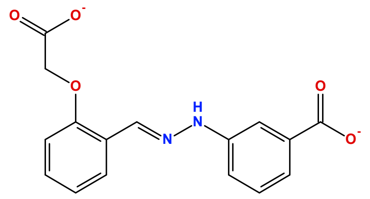

Docking Score: -5.0618

ID: 43

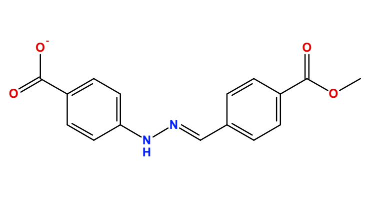

Docking Score: -5.7904

ID: 44

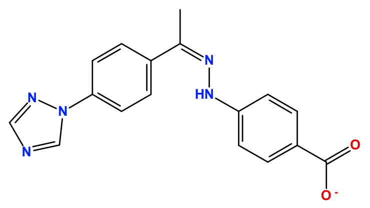

Docking Score: -4.8733

ID: 45

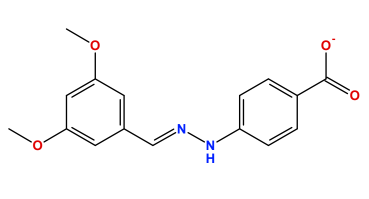

Docking Score: -6.8587

ID: 52

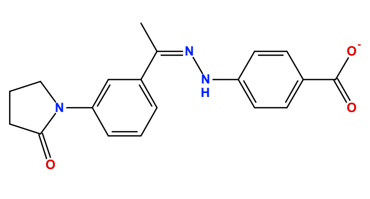

Docking Score: -4.4183

ID: 53

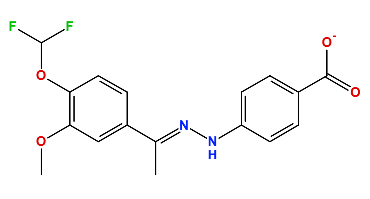

Docking Score: -6.3671

ID: 54

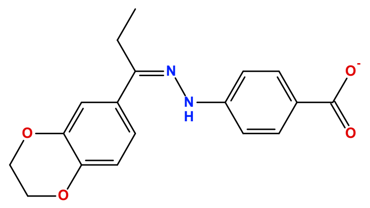

Docking Score: -4.3787

ID: 57

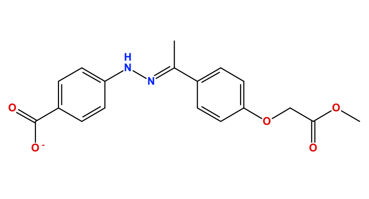

Docking Score: -5.2187

ID: 58

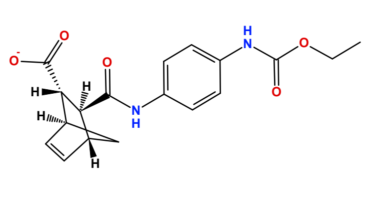

Docking Score: -6.5797

ID: 66

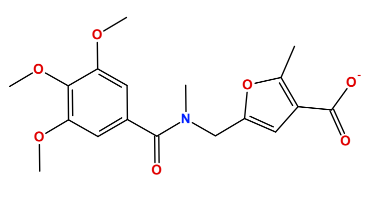

Docking Score: -6.3110

ID: 70

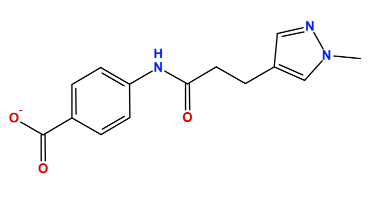

Docking Score: -6.3887

ID: 71

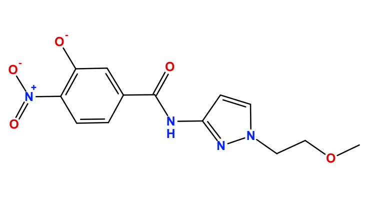

Docking Score: -4.6766

ID: 72

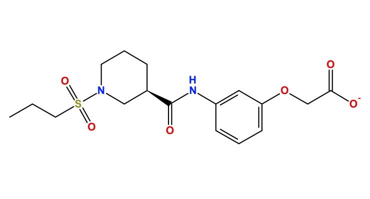

Docking Score: -8.7156

ID: 105

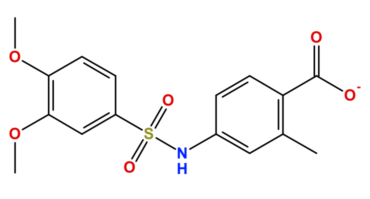

Docking Score: -6.7091

ID: 107

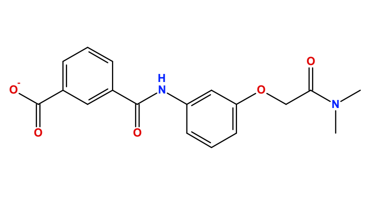

Docking Score: -7.0617

ID: 109

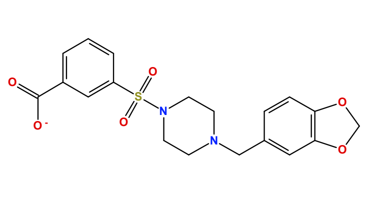

Docking Score: -7.2678

ID: 110

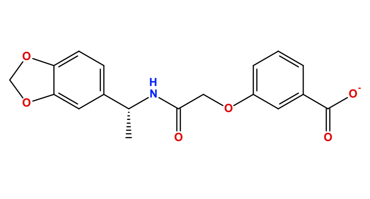

Docking Score: -6.5004

ID: 117

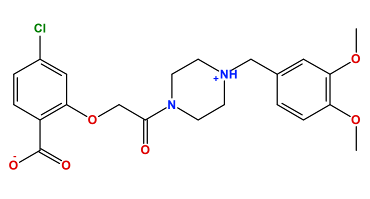

Docking Score: -5.8271

ID: 118

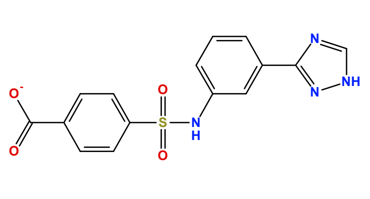

Docking Score: -5.1635

ID: 119

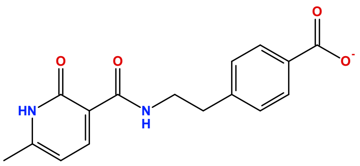

Docking Score: -5.4642

ID: 124

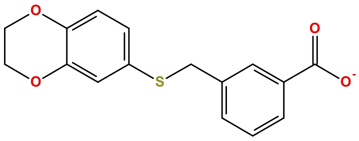

Docking Score: -4.2861

ID: 127

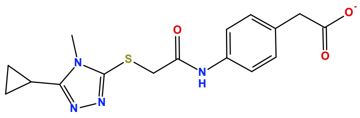

Docking Score: -5.6401

ID: 129

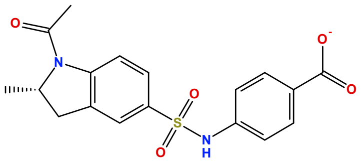

Docking Score: -5.9969

ID: 140

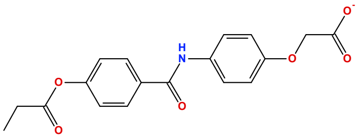

Docking Score: -6.8620

ID: 142

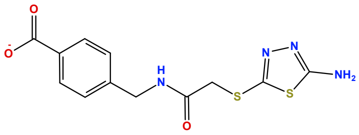

Docking Score: -5.0773

ID: 151

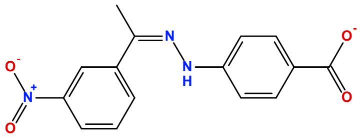

Docking Score: -5.0761

ID: 155

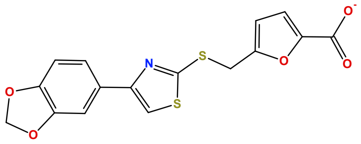

Docking Score: -8.3982

ID: 156

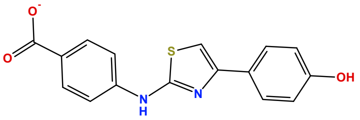

Docking Score: -6.2560

ID: 158

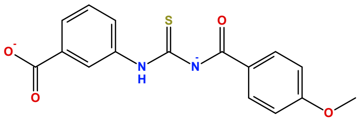

Docking Score: -6.1802

ID: 162

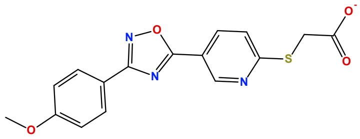

Docking Score: -9.0565

ID: 163

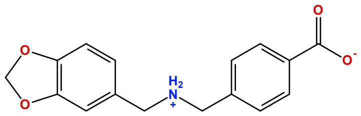

Docking Score: -5.2356

ID: 165

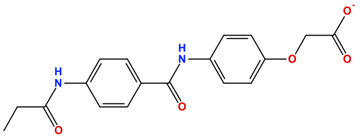

Docking Score: -6.7105

ID: 166

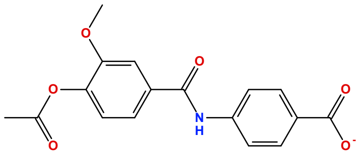

Docking Score: -7.4558

ID: 169

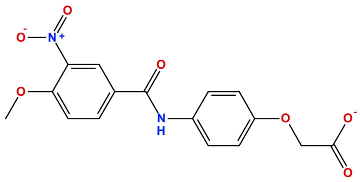

Docking Score: -8.5761

ID: 170

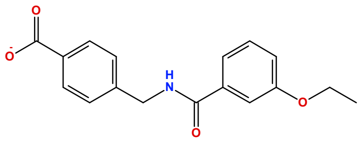

Docking Score: -5.5946

ID: 171

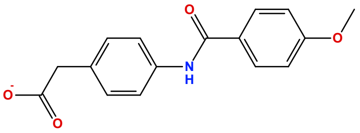

Docking Score: -6.8541

ID: 174

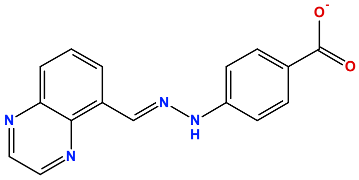

Docking Score: -6.5208

ID: 177

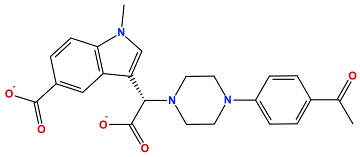

Docking Score: -6.5813

ID: 178

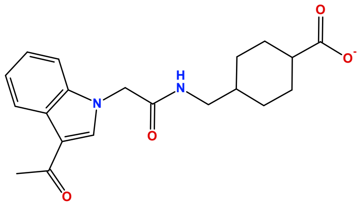

Docking Score: -6.1561

ID: 206

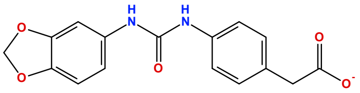

Docking Score: -5.1196

ID: 208

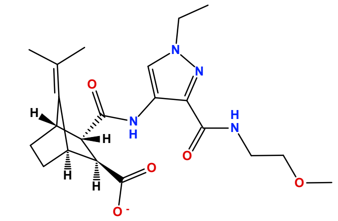

Docking Score: -4.2659
